# Combined femoral and popliteal nerve block is superior to local periarticular infiltration anaesthesia for postoperative pain control after total knee arthroplasty

**DOI:** 10.1007/s00167-022-06868-w

**Published:** 2022-02-03

**Authors:** Gregor A. Schittek, Patrick Reinbacher, Martin Rief, David Gebauer, Andreas Leithner, Ines Vielgut, Viktor Labmayr, Holger Simonis, Markus Köstenberger, Helmar Bornemann-Cimenti, Andreas Sandner-Kiesling, Patrick Sadoghi

**Affiliations:** 1 Department of Orthopaedics and Trauma, Medical University of Graz, Auenbruggerplatz 5, 8036 Graz, Austria; 2 Department of Anaesthesia and Intensive Care, Medical University of Graz, Graz, Austria

**Keywords:** Postoperative pain, Opioid sparing, Total knee replacement, Local infiltration anaesthesia, Ultrasound-guided regional nerve blocks, Dexmedetomidine

## Abstract

**Introduction:**

After primary total knee arthroplasty (TKA), local periarticular infiltration anaesthesia (LIA) is a fast and safe method for postoperative pain control. Moreover, ultrasound-guided regional anaesthesia (USRA) with femoral and popliteal block is a standard procedure in perioperative care. Two analgesic regimens for TKA—LIA versus URSA with dexmedetomidine—were compared as an additive to ropivacaine. We hypothesised that the use of URSA provides a superior opioid sparing effect for TKA compared with LIA.

**Methods:**

Fifty patients (planned 188 participants; safety analysis was performed after examining the first 50 participants) were randomised. These patients received LIA into the knee capsule during surgery with 60 ml of ropivacaine 0.5% and 1 ml of dexmedetomidine (100 µg ml^−1^) or two single-shot URSA blocks (femoral and popliteal block) before surgery with 15 ml of ropivacaine 0.5% and 0.5 ml of dexmedetomidine for each block. Postoperative opioid consumption in the first 48 h, pain assessment and complications were analysed.

**Results:**

In the safety analysis, there was a significantly higher need for opioids in the LIA group, with a median oral morphine equivalent of 42.0 [interquartile range (IQR) 23.5–57.0] mg versus 27.0 [IQR 0.0–33.5] mg (*P* = 0.022). Due to this finding, the study was terminated for ethical considerations according to the protocol.

**Conclusion:**

This is the first study presenting data on LIA application in combination with dexmedetomidine. A superior opioid-sparing effect of URSA was observed when compared with LIA in TKA when dexmedetomidine is added to local anaesthetics. Also, a longer lasting opioid-sparing effect in the LIA group was observed when compared with the recently published literature; this difference could be attributed to the addition of dexmedetomidine. Therefore, multimodal analgesia regimens could be further improved when LIA or USRA techniques are combined with dexmedetomidine.

## Introduction

Pain intensity after total knee arthroplasty (TKA) peaks the first days after surgery. Pain affects the rehabilitation progress negatively and leads to prolonged hospitalisation [7, 8, 13, 22]. Pain negatively influences recovery and rehabilitation because it can negatively interfere with rehabilitation training and affect the patient’s activity levels, thus possibly leading to prolonged hospitalisation [4]. Recently, there has been an increased focus on multimodal pain management such as targeting multiple pain pathways, combining opioids, providing nonsteroidal anti-inflammatory drugs and periarticular injections or ultrasound-guided regional anaesthesia (USRA) [25]. The aim of these multimodal analgesic regimens is to reduce the need of anaesthetics and opioids while improving the patient’s comfort and recovery.

Local infiltration anaesthesia (LIA), also known as periarticular injections, during TKA is one possible method in a multimodal pain management to reduce pain postoperatively [2, 5, 19]. A meta-analysis revealed that pain was lower in patients with LIA at 6 and 24 h postoperatively compared with placebo [10]. A further argument for the use of LIA is the simple and fast application with a minimal side effect profile such as a reduced risk of potential neurological damage and muscle weakness, associated with peripheral regional anaesthesia [3, 14]. Many authors prefer LIA because of the problem of reduced patient motor function after surgery and femoral nerve block [24].

The most common targets for postoperative analgesia by USRA in TKA are the femoral, sciatic and obturator nerves. Patients with single-shot femoral nerve block have a significantly lower need for opioids during the first 48 h postoperatively compared with placebo [18]. A block of the sciatic nerve in the popliteal region, in addition to a blockade of the femoral nerve, is suggested by some authors to achieve better analgesia [1]. Furthermore, the block duration can be increased by using adjuvants such as dexmedetomidine with local anaesthetics [14]. Dexmedetomidine is a α2-adrenoceptor agonist with sympatholytic, analgesic and sedative effects. It is widely used as a local anaesthetic adjuvant due to its block-prolonging effect when a single shot of USRA is performed [27].

To date, the potential of dexmedetomidine as an adjuvant to LIA has not been investigated. If a dexmedetomidine-supplemented LIA could similarly enhance the analgesic efficacy of ropivacaine and prolong the duration of postoperative analgesia after TKA, as has already been described for wound infiltration, then this would be another strong argument for the use of LIA instead of USRA [15, 21]. Thus, the primary goal of the present study was to evaluate dexmedetomidine as an adjuvant to LIA in TKA, when compared to the effect of single-shot femoral and popliteal blocks, both combined with dexmedetomidine, on postoperative opioid consumption. The hypothesis was that ultrasound-guided nerve blockades are superior to LIA in relation to postoperative pain control.

## Methods

The study was performed from February to April 2021 at a university hospital. All adult patients scheduled for primary TKA for severe osteoarthritis (OA) of the knee and who consented to participate (gave written informed consent) were eligible for study participation. Exclusion criteria were pregnancy, breastfeeding and allergies to the study medications.

Patients were randomly assigned to either the LIA group or the USRA group in a 1:1 ratio. The allocation sequence was generated before surgery by using a web-based randomisation tool of the Institute for Medical Informatics, Statistics and Documentation of the local university. (https://www.randomizer.at). Patients and treating physicians were aware of the group assignment. In the LIA group, at the end of the surgery the surgeon performed the procedure as described below. In the USRA group, the patients received two ultrasound-guided peripheral nerve blocks by their anaesthesiologist shortly before surgery.

### Local infiltration anaesthesia procedures and regional anaesthesia

In the LIA group, patients received periarticular infiltration with 60 ml of ropivacaine 0.5% and 1 ml of dexmedetomidine (100 μg ml^−1^) around the knee joint including the posterior capsule to block distal nerve fibres. Infiltration was performed after the implantation of the femoral and tibial components before positioning the liner. Consequently, the infiltration procedure addressed the knee joint capsule plus the posterior joint structures, periarticular soft tissue and subcutaneous soft tissue before skin closure and end of surgery.

In the USRA group, both single-shot peripheral nerve blocks were conducted just before induction of general anaesthesia or spinal anaesthesia according to the local standard operating procedure. Under sterile conditions, a 120 mm 22-gauge needle (Pajunk SonoplexStim; GmbH Medizintechnologie, Geisingen, Germany) was used to perform the blocks. A linear ultrasound transducer (frequency 10–12 MHz) was used to visualise the target nerves, needle and surrounding structures.

The distal single-shot popliteal nerve block was performed approximately 1–3 cm before the division of the sciatic nerve into the tibial nerve and the common peroneal nerve, with a safe distance to the popliteal crease. This nerve block was performed with the patient in the supine position resting their foot on an elevated foot rest. An ultrasound-guided in-line needle insertion technique was used for needle placement and control of local anaesthetic spreading. A mixture containing 15 ml of ropivacaine 0.5% and 0.5 ml of dexmedetomidine (100 μg ml^−1^) was injected perineurally. Subsequently, an ultrasound-guided single-shot femoral nerve block was performed before anaesthesia induction with concurrent intravenous administration of remifentanil (for both blockades) to reduce the patient’s discomfort during placement of regional anaesthesia [23]. Patients were placed in the supine position with the table flattened to access the inguinal area. An ultrasound-guided in-line needle insertion technique was used for correct needle placement and a mixture containing 15 ml of ropivacaine 0.5% and 0.5 ml of dexmedetomidine (100 μg ml^−1^) was injected perineurally. USRA was performed of an anaesthetist.

### Total knee arthroplasty

All patients received a single-shot antibiotic prophylaxis preoperatively (2 g of cefazolin intravenously approximately 30 min before skin incision). No wound drains were used in the patients and no postoperative antibiotics were administered.

The TKAs were carried out by one senior knee surgeon using the same surgical approach with no patellar resurfacing in an extension gap first, namely the flexion gap balanced technique. Femoral and tibial implants were cemented (Palacos R + G, Heraeus Medical, Wehrheim, Germany). All participants received an Attune TKA (DePuy, Synthes, Warsaw, IN, USA). All the patients had been experiencing severe OA of the knee—the diagnosis had been confirmed by radiography (Kellgren–Lawrence-Score III/IV)—with persistent pain in at least two knee compartments despite conservative treatment and reduced knee mobility. Postoperatively, patients followed a standardised rehabilitation protocol consisting of full weight bearing immediately after surgery including the use of crutches and a continuous passive motion (CPM) therapy on day one after surgery. There were no differences observed between the groups.

### Additional anaesthetic technique

In addition to LIA or USRA, either spinal anaesthesia or general anaesthesia was performed. To avoid a possible carry-over effect with the general anaesthetic technique, patients were treated only with remifentanil during general anaesthesia so as not to distort the pain perception in the early recovery period by long-acting opiates.

### Postoperative treatment

Postoperatively, all patients were treated with fixed doses of non-opioid oral analgesics (600 mg of ibuprofen) three times daily according to the local standard operating procedure. In addition, all patients received opioids (piritramide) at the end of the surgery and by the ward nurses as needed for pain control. Pain was measured four times daily by using a self-rating 11-point Numerical Rating Scale (NRS) from 0 (no pain) to 10 (worst pain imaginable) at rest and when doing exercises (free movements of the operated extremity). Patients received opioids (piritramide 2.5–3 mg intravenous bolus, 10 min until re-evaluation) until the self-rating NRS was specified below 4 at rest and below 5 when doing exercises.

### Study endpoints

The primary endpoint for this randomised controlled trial was postoperative opioid consumption calculated in oral morphine equivalents (OME) needed during the first 48 h after TKA for pain control. NRS pain scores were documented at rest and during exercise. Data collection was performed by physicians blinded to the patient’s anaesthetic treatment. Pain was evaluated at rest and during exercise by using the NRS four times daily. Daily oral morphine equivalent (OME) consumption was documented until hospital discharge. To calculate OME, we used the following ratios: 1 mg morphine intravenous = 1.5 mg piritramide intravenous = 3 mg morphine oral [6, 12].

It has to be highlighted that all the patients scheduled for TKA in our hospital, regardless of this study, are admitted 1–2 days preoperatively and treated 5 days postoperatively as inpatients. For this reason, we chose ‘complications’ rather than ‘treatment duration’ as a secondary outcome.

Each patient’s medical history such as treatment data like opioid consumption and NRS were recorded by the in-house electronic documentation system (SAP Open Medocs, SAP SE, Walldorf, Germany and EDV GmbH, Debis Systemhaus) of the hospital.

### Statistical analysis

Data are reported as the number of patients (%), mean ( ± standard deviation [SD]) for parametric data or median (25th to 75th percentiles, known as the interquartile range [IQR]) for non-parametric data. The following tests were performed: the Kolmogorov–Smirnov and Shapiro–Wilk tests to test the normality of the data; Fisher’s exact test for univariate analyses of statistical significance or the Mann–Whitney test for nonparametric data. Statistical significance was analysed with a two-sided alpha of < 5% set as the significance level. In exploratory analyses of the data, an adjusted *P* value (*P*2) of < 0.01 was applied to account for multiple testing. Further analyses included rank correlation with Spearman's ρ and logistic regression. In detail, to assess a possible correlation between LIA use and OME requirements or NRS scores (at rest and during exercise), we performed Spearman correlations. The logistic regression models for opioid consumption in OME and postoperative pain were ‘type of anaesthesia’ (spinal anaesthesia [binary]), ‘type of administration of local anaesthetics’ (LIA [binary]) and ‘sex’ (binary). In this logistic regression analysis, we dichotomised the NRS with a threshold of < 3 or > 3 on the second postoperative day and the inequality in administered OME during the first 48 h compared with the median of the USRA group (greater or same/less). We chose the NRS on the second postoperative day because on this day the effect of local anaesthetics has definitely has worn off.

A safety analysis after enrolling 50 participants was executed by the responsible physicians in accordance with the consultation with the local ethics committee board. A clinically meaningful difference in opioid consumption between the groups was considered to be > 10 mg OME needed for postoperative pain control. This manuscript adheres to the applicable CONSORT guidelines. An a priori power analysis (Statistical Solutions Ltd nQuery Advisor Version 8.4.1 2019; Cork, Ireland) was conducted based on observations in prior knee arthroplasty studies. A total sample size of 188 (176 plus drop-out) patients was calculated to detect a significant difference of 13.2 mg OME in the primary endpoint of median opioid equivalents consumption 48 h after surgery between the study groups (alpha 5%, Kruskal–Wallis test, 1 – β probability of 80%).

### Institutional review board

This randomised controlled clinical trial was approved by the institutional review board of the Medical University (Ethical Committee No. 32-239 ex 19/20, Chairperson: on 16 December 2020, Medical University) and registered with data safety authorities. All study patients provided written informed consent to participate in the trial.

### Clinical trial registration

The study was registered at Clinicaltrails.gov.

## Results

**Fig. 1:**
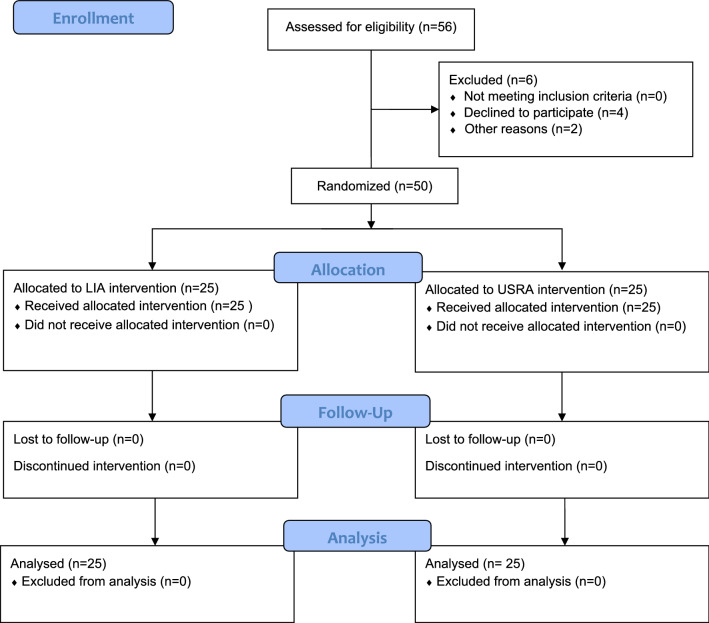
Fifty-six consecutive patients were screened for eligibility, and 50 were randomised and given local periarticular infiltration anaesthesia (LIA) into the knee capsule during surgery with 60 ml of ropivacaine 0.5% and 1 ml of dexmedetomidine (100 μg ml^−1^) or two single-shot ultrasound-guided regional anaesthesia (USRA) blocks (femoral and popliteal block) before total knee arthroplasty (TKA) with 15 ml of ropivacaine 0.5% and 0.5 ml of dexmedetomidine for each block

Fifty-six consecutive patients were screened for eligibility (Fig. 1). Of these, four declined to participate and two other patients were rescheduled for later TKA surgery after screening. Therefore, 50 patients were included in this trial. Dropout and complication rates in this study were 0%. The complication time frame for dexmedetomidine as part of the clinical trial was 5 days.

There was no difference between the two groups regarding the baseline characteristics of the participating patients (Table 1). Only the applied main anaesthetic technique (general anaesthesia was used more frequently in the USRA group, *P* = 0.037) differed significantly.

**Table 1:** Baseline characteristics of 50 patients undergoing primary total knee arthroplasty with general or spinal anaesthesia randomised to receive additionally local infiltration anaesthesia or ultrasound guide regional anaesthesia for postoperative pain control

	LIA, *N* = 25	USRA, *N* = 25	*P*
Age (years)	68.6 ± 10.2	67.6 ± 11.0	0.771
Female	12 (48)	10 (40)	0.569
Height	167.1 ± 9.4	171.0 ± 12.1	0.438
Weight	77.0 [70.0 to 88.5]	85.0 [70.0 to 131.2]	0.437
BMI (kg m^−2^)	28.4 [25.7 to 31.6]	27.8 [24.3 to 33.8]	0.734
ASA physical status 1	0 (0)	1 (4)	
2	10 (40)	7 (28)	0.437
3	15 (60)	17 (68)	
Main anaesthetic technique			
General anaesthesia	5 (20)	11 (44)	0.037
Spinal anaesthesia	20 (80)	14 (56)	
Days of hospitalisation	6.0 [6.0 to 7.0]	6.0 [6.0 to 7.0]	0.639

Values are number of patients (%), mean ± SD or median [IQR]

*LIA* local periarticular infiltration anaesthesia technique, *USRA* ultrasound guide regional anaesthesia, *ASA* physical status classification system by the American Society of Anaesthesiologists

*For multiple testing an adjusted significance value of *P*_*2*_ < 0.01 is applicable

A significantly higher opioid consumption was observed in the LIA group (LIA group 42.0 [IQR 24.0–57.0] mg versus USRA group 27.0 [IQR 0.0–34.0] mg, *P* = 0.022) during the first 48 h after TKA surgery. The difference of 15 mg OME between the groups lead to study termination by the responsible study physician for ethical considerations (in accordance with the protocol approved by the institutional review board).

Despite the more frequent use of spinal anaesthesia in the LIA group, opioid consumption in the postanaesthesia care unit (PACU) was significantly higher (LIA group 12.0 [IQR 10.0–20.5] mg versus USRA group 0.0 [IQR 0.0–18.0] mg, *P* = 0.047) (Table 2). The main anaesthetic technique (general vs spinal anaesthesia) differed but does not seem to have influenced the results according to logistic regression. The need for opioids on the ward on the surgery day after the patient was discharged by the PACU physicians was also higher in the LIA group (15.0 [IQR 0.0–15.0] mg versus 0.0 [IQR 0.0–0.0] mg, *P* < 0.001). Thus, the combined need for opioids in the PACU and the ward on the surgery day was higher in the LIA group (27.0 [IQR 16.5–35.5] mg versus 3.0 [IQR 0.0–23.5] mg, *P* = 0.001). Forty-eight hours after TKA and onwards, no statistical difference could be found between the study groups, mainly because the patients no longer required opioids—for example, on day 2, LIA group median 0 [IQR 0–0] mg versus USRA group median 0 [IQR 0.0–15] mg (*P* = 0.768).

**Table 2:** Postoperative oral morphine equivalents (OME) in 50 patients undergoing primary total knee arthroplasty with general or spinal anaesthesia randomised to receive additionally local infiltration anaesthesia or ultrasound guide regional anaesthesia for postoperative pain control

	LIA, *n* = 25	USRA, *n* = 25	*P*
OME in the PACU	12.0 [10.0 to 20.5]	0.0 [0.0 to 18.0]	0.047
OME on the ward Day 0	15.0 [0.0 to 15.0]	0.0 [0.0 to 0.0]	< 0.001
OME in PACU + ward (Day 0)	27.0 [16.5 to 35.5]	3.0 [0.0 to 23.5]	0.001
OME 48 h post TKA (PACU, ward Day 0, Day 1)	42.0 [23.5 to 57.0]	27.0 [0 to 33.5]	0.022
OME on the ward*			
Day 1	15.0 [0 to 15.0]	0.0 [0.0 to 22.5]	0.570
Day 2	0.0 [0 to 15.0]	0.0 [0.0 to 22.5]	0.768
Day 3	0.0 [0.0 to 0.0]	0.0 [0.0 to 15.0]	0.564
Day 4	0.0 [0.0 to 0.0]	0.0 [0.0 to 0.0]	0.267
Day 6	0.0 [0.0 to 0.0]	0.0 [0.0 to 0.0]	0.977

Values are median [IQR]

*OME* oral morphine equivalents, *LIA* local periarticular infiltration anaesthesia technique, *USRA* ultrasound guide regional anaesthesia, *PACU* post-anaesthesia care unit; oral morphine equivalents in mg

*For multiple testing of opioid does, an adjusted significance value of *P*_*2*_ < 0.01 is applicable

Significantly higher NRS scores during exercise were observed in the LIA group on the first postoperative day (Table 3; LIA group 2.0 [1.0–4.0] versus USRA group 0.0 [0–2.0]; *P*2 = 0.001). Further, the NRS scores in the PACU and on the second postoperative day were not significantly different. No meaningful difference between the study groups could be found after the second postoperative day. There were no adverse effects of dexmedetomidine or other complications in either study group during the perioperative period or during postoperative visits.

**Table 3:** Postoperative maximum numeric pain scores (NRS) at rest and during exercise in 50 patients undergoing primary total knee arthroplasty with general or spinal anaesthesia randomised to receive additionally local infiltration anaesthesia or ultrasound guide regional anaesthesia for postoperative pain control

	LIA, *n* = 25	USRA, *n* = 25	P*
Maximum NRS at rest in/at			
PACU	0.0 [0.0 to 1.8]	0.0 [0.0 to 0.5]	0.091
Day 1	1.0 [0.0 to 3.0]	0.0 [0.0 to 0.5]	0.006
Day 2	2.0 [1.0 to 4.0]	1.0 [0.0 to 3.5]	0.313
Day 3	1.0 [0.0 to 2.0]	1.0 [0.0 to 1.0]	0.621
Day 4	0.0 [0.0 to 1.0]	0.0 [0.0 to 1.0]	0.723
Day 5	1.0 [0.0 to 1.0]	0.5 [0.0 to 1.0]	0.907
Day 6	0.0 [0.0 to 1.5]	1.5 [0.0 to 3.0]	0.267
Maximum NRS during exercise in/at			
PACU	0.0 [0.0 to 2.8]	0.0 [0.0 to 1.0]	0.011
Day 1	2.0 [1.0 to 4.0]	0.0 [0.0 to 2.0]	0.001
Day 2	2.0 [2.0 to 5.0]	2.0 [1.0 to 5.0]	0.282
Day 3	2.0 [1.0 to 3.0]	2.0 [1.0 to 2.5]	0.912
Day 4	1.0 [0.5 to 2.0]	1.0 [0.5 to 2.5]	0.873
Day 5	1.0 [0.5 to 2.0]	2.0 [1.0 to 2.0]	0.345
Day 6	1.0 [1.0 to 4.0]	2.5 [1.0 to 4.0]	0.236

Values are median [IQR]

*LIA* local periarticular infiltration anaesthesia technique, *USRA* ultrasound guide regional anaesthesia, *PACU* post-anaesthesia care unit, *units in NRS* numerical rating scale

*Please note that for multiple testing of NRS values, an adjusted significance value of *P*_2_ < 0.01 is applicable

A weak correlation between LIA and the OME needed (*r* = 0.327, *P* = 0.02) as well as for pain during exercise in the PACU (*r* = 0.312, *P* = 0.03) could be identified. There were moderate correlations between LIA and the OME in the PACU as well as between LIA and pain experienced during exercise on the ward on the first postoperative day (*r* = 0.511 and *r* = 0.476, respectively, *P* < 0.001). The logistic regression adjusted for the type of anaesthesia (spinal anaesthesia), the type of LIA and sex were all non-significant (due to the 95% confidence interval including the odds ratio of 1.0) (Table 4). No episodes of bradycardia or decrease of blood pressure after anaesthesia induction or LIA that had to be treated by medication (atropine or ephedrine) were detected in either group.

**Table 4:** Logistic regression on opioid consumption during the first 48 h postoperatively and pain during exercise on the second postoperative day

Opioid consumption	OR	95% CI	*P* value
Spinal anaesthesia	1.014	0.316 to 3.728	0.963
LIA	2.511	0.768 to 8.205	0.128
Male sex	1.086	0.316 to 3.728	0.896
Pain			
Spinal anaesthesia	1.121	0.628 to 1.999	0.699
LIA	1.626	0.506 to 5.232	0.415
Male sex	0.782	0.234 to 2.613	0.69

The logistic regression model fitted with the covariates “type of anesthesia” (spinal anaesthesia), “type of administration of local anaesthetics “(LIA) and “sex” (male sex). Tendency below 1 in OR stands for lower need of opioids and less pain

*OR* odds ratio, *CI* confidence interval

## Discussion

This is the first study presenting data on LIA application in combination with dexmedetomidine. The most important finding of the present study is that the opioids needed in the first 48 h after surgery in the USRA group were significantly lower, a finding that indicates better early postoperative pain control after primary TKA. However, it has to be highlighted that the observed opioid consumption in the LIA group of 42 mg OME (14 mg morphine intravenous in 48 h) is lower than in any study described in a recent review [20]. Furthermore, it could be shown that the maximum NRS scores were lower in the USRA group from day 0 to day 2 after TKA, reaching a significant difference on day 1 (Table 3). These results contradict the outcomes of previous studies describing LIA as an equivalent technique to USRA for postoperative pain management [11, 26, 28]. In detail, a randomised trial including 166 patients showed that the use of LIA compared with femoral nerve catheter with additional single-shot sciatic nerve block resulted in similar postoperative pain reduction. On the other hand, patients who received LIA needed a higher OME (12 mg vs 5 mg) on the day of surgery [26]. A higher rate of neural injuries was reported in the USRA group (9 vs 1 patients) 6 weeks after surgery (follow-up according to the study protocol) [26]. In another randomised controlled trial published in 2019, Kastelik et al. [11] demonstrated that the use of LIA compared with the adductor canal block/catheter with additional single-shot popliteal nerve block resulted in similar postoperative pain reduction and even OME. Only the reported time of anaesthesia induction differed significantly: on average, it was 25 min longer in the USRA group [11]. A retrospective cohort study published in 2020 with 206 patients receiving either LIA or a combination of single-shot femoral and popliteal nerve blocks showed no differences between the study groups in postoperative pain or opioid use [28].

It must be noted once again that in the above-mentioned studies, the authors reported postoperative opioid doses in OME that are higher than in this trial. For example, Kastelik et al. [11] reported much higher total OME than in this study for both the LIA and USRA groups (160 [LIA group] and 165 [USRA group] mg vs 42 [LIA group] and 27 [USRA group]). In addition to the opioid consumption, the reported maximum NRS pain scores in this study were also lower than those by Kastelik et al. [11]. Thus, considering the current literature, these results suggest a possible advantage when combining ropivacaine and dexmedetomidine. Unfortunately, for the current clinical trial the local ethics committee rejected the originally planned LIA control group without dexmedetomidine. Thus, it can only be speculated about an improved efficiency of LIA combined with dexmedetomidine.

In our opinion, this study shows stronger and longer lasting postoperative pain control in connection with USRA than with LIA after TKA. However, maximum postoperative pain control may have a price. Due to the problem of reduced patient motor function after surgery and femoral nerve block, many authors prefer LIA [24]. This is also underlined by the fact that the OME and NRS we have reported are much lower than in comparable studies.

In this study, the focus was set on postoperative opioid consumption and pain experienced. The time required to perform LIA or USRA was not measured but only the total time operation time, which did not significantly differ between groups. Some authors have stated that USRA is much more time-consuming compared with the easy-to-perform intraoperative LIA technique, resulting in fewer possible TKAs on a surgery day than usual when performing USRA [9, 11, 16, 17]. In addition, LIA appears to be a more cost-effective technique [21]. Regarding adverse effects of dexmedetomidine for LIA or USRA, we no evidence for the concentration used could be found.

Regarding the limitations of this study, it must be point out that the study design (single centre, missing control groups without dexmedetomidine) complicates the interpretation of the results. Second, the main anaesthetic technique (general vs spinal anaesthesia) differed but does not seem to have influenced the results according to logistic regression. Third, ketorolac was not used in order to enable a comparison of the effect of dexmedetomidine. Furthermore, an unusual volume of ropivacaine was used for LIA by decision of the lead surgeon. Due to logistic aspects of the local health care system, the length of stay (LOS) after TKA in Austria is comparatively long compared with international studies. In future studies, the anaesthetic technique (either spinal or general anaesthesia) should be considered during planning to mitigate the potential bias of a large difference. USRA was mainly performed by specialists of anaesthesia; when a resident performed the block, a specialist was present to supervise the resident. As described in the current literature, dexmedetomidine plays a role as an additive to the local anaesthetics. Because the initially planned control group for dexmedetomidine was not permitted by the local ethics only a comparison with other studies was possible in order to compare the effect of a LIA without dexmedetomidine to a LIA with it.

## Conclusion

A superior opioid-sparing effect of URSA was observed when compared with LIA in TKA when dexmedetomidine is added to local anaesthetics. Also, a longer lasting opioid-sparing effect in the LIA group was observed when compared with the recently published literature; this difference could be attributed to the addition of dexmedetomidine. Therefore, multimodal analgesia regimens could be further improved when LIA or USRA techniques are combined with dexmedetomidine. Additional studies are necessary to determine recommendations concerning the ideal concentrations of ropivacaine and the amount of dexmedetomidine.
